# The Arabidopsis TOR Kinase Specifically Regulates the Expression of Nuclear Genes Coding for Plastidic Ribosomal Proteins and the Phosphorylation of the Cytosolic Ribosomal Protein S6

**DOI:** 10.3389/fpls.2016.01611

**Published:** 2016-11-07

**Authors:** Thomas Dobrenel, Eder Mancera-Martínez, Céline Forzani, Marianne Azzopardi, Marlène Davanture, Manon Moreau, Mikhail Schepetilnikov, Johana Chicher, Olivier Langella, Michel Zivy, Christophe Robaglia, Lyubov A. Ryabova, Johannes Hanson, Christian Meyer

**Affiliations:** ^1^Institut Jean-Pierre Bourgin, Institut National de la Recherche Agronomique, AgroParisTech, Centre National de la Recherche Scientifique, Université Paris-SaclayVersailles, France; ^2^Université Paris-Sud–Université Paris-SaclayOrsay, France; ^3^Umeå Plant Science Center, Department of Plant Physiology, Umeå UniversityUmeå, Sweden; ^4^Institut de Biologie Moléculaire des Plantes, UPR 2357 CNRS, Université de StrasbourgStrasbourg, France; ^5^Plateforme PAPPSO, UMR GQE-Le MoulonGif sur Yvette, France; ^6^Laboratoire de Génétique et Biophysique des Plantes, UMR 7265, DSV, IBEB, SBVME, CEA, CNRS, Aix-Marseille Université, Faculté des Sciences de LuminyMarseille, France; ^7^Plateforme Protéomique Strasbourg-Esplanade, CNRS FRC1589, Institut de Biologie Moléculaire et CellulaireStrasbourg, France

**Keywords:** phosphorylation, plastid, proteomic, ribosome, RPS6, TOR kinase, transcriptomic, translatomic

## Abstract

Protein translation is an energy consuming process that has to be fine-tuned at both the cell and organism levels to match the availability of resources. The target of rapamycin kinase (TOR) is a key regulator of a large range of biological processes in response to environmental cues. In this study, we have investigated the effects of TOR inactivation on the expression and regulation of Arabidopsis ribosomal proteins at different levels of analysis, namely from transcriptomic to phosphoproteomic. TOR inactivation resulted in a coordinated down-regulation of the transcription and translation of nuclear-encoded mRNAs coding for plastidic ribosomal proteins, which could explain the chlorotic phenotype of the TOR silenced plants. We have identified in the 5′ untranslated regions (UTRs) of this set of genes a conserved sequence related to the 5′ terminal oligopyrimidine motif, which is known to confer translational regulation by the TOR kinase in other eukaryotes. Furthermore, the phosphoproteomic analysis of the ribosomal fraction following TOR inactivation revealed a lower phosphorylation of the conserved Ser240 residue in the C-terminal region of the 40S ribosomal protein S6 (RPS6). These results were confirmed by Western blot analysis using an antibody that specifically recognizes phosphorylated Ser240 in RPS6. Finally, this antibody was used to follow TOR activity in plants. Our results thus uncover a multi-level regulation of plant ribosomal genes and proteins by the TOR kinase.

## Introduction

During their life, living organisms have to adapt their growth and development to exogenous factors such as stresses and nutrient availability. Therefore, they have evolved different regulatory pathways to increase the perception of environmental cues and to fasten the required metabolic modifications. These pathways employ conserved key players that link energy depletion, which is often the result of stresses and nutrient limitation, to anabolic and catabolic cellular activities. One of the most important pathway that is found in all eukaryotes is the one related to the target of rapamycin (TOR) protein kinase. TOR is a large kinase, which operates in at least two multi-protein complexes (TORC1 and TORC2; for reviews, see: Wullschleger et al., 2006; Laplante and Sabatini, 2012; Albert and Hall, 2015) and controls a wealth of biological outputs. In animals and yeast, it is well known that TOR positively regulates protein synthesis and anabolic activities when the growth conditions are favorable, while repressing the mechanisms implicated in recycling and catabolism (Laplante and Sabatini, 2012; Shimobayashi and Hall, 2014). Indeed, the production of proteins is particularly energy consuming since it requires ribosome biogenesis as well as mRNA translation (Warner, 1999).

In plants, there is so far only evidence for the presence of the TORC1 complex which comprises the conserved Regulatory-associated protein of TOR (RAPTOR) and the Lethal with Sec 13 (LST8) proteins, (for reviews, see: Robaglia et al., 2012; Henriques et al., 2014; Xiong and Sheen, 2014; Rexin et al., 2015; Dobrenel et al., 2016). TOR has already been shown to control a vast array of biological processes in plants (Deprost et al., 2007; Caldana et al., 2013; Xiong and Sheen, 2014; Dong et al., 2015) and a link between the TOR complex and protein translation has been evidenced. We have indeed shown earlier that TOR inactivation, either after silencing (Deprost et al., 2007) or by using a TOR inhibitor (Sormani et al., 2007) leads to a decrease in polysome abundance. It has also been shown that the translation reinitiation after a long upstream open reading frame by the plant viral reinitiation factor transactivator-viroplasmin is mediated by its physical association with the TOR protein (Schepetilnikov et al., 2011). Furthermore, TOR activity appears essential for translation reinitiation of cellular mRNA containing short-ORFs in the 5′ UTR (Schepetilnikov et al., 2013).

The biochemical analysis of the plant cytoplasmic ribosome showed that it contains 81 different proteins, 33 for the small 40S subunit and 48 for the large 80S subunit (Giavalisco et al., 2005; Carroll et al., 2008; Carroll, 2013; Browning and Bailey-Serres, 2015; Hummel et al., 2015). In animal cells, TOR regulates cap-dependent translation by phosphorylating and stimulating the activity of the ribosomal protein S6 kinase (S6K), a conserved target of TOR which phosphorylates the 40S ribosomal protein S6 (RPS6, Barbet et al., 1996; Holz et al., 2005), and by repressing the inhibitory effect of eIF4E-binding protein (Ma and Blenis, 2009; Albert and Hall, 2015). Consistently, in yeast, TOR inhibition by rapamycin leads to an 80% reduction in overall translation (Barbet et al., 1996). It has been shown in mammals that S6K is activated in a TOR-dependent manner by phosphorylation of Thr389 and Thr229 (Ma and Blenis, 2009), resulting subsequently in the phosphorylation of serine residues in the C-terminal extremity of the ribosomal protein RPS6. Early on RPS6, which is located at the right foot of the 40S subunit, was identified as the only phosphorylated protein in the ribosome small subunit (Gressner and Wool, 1976).

It has been postulated that TOR regulates the translation of a particular sub-set of mRNAs containing a 5′ terminal tract oligopyrimidine (TOP) motif (for a review, see Meyuhas and Kahan, 2015). Canonical TOP mRNAs harbor a C residue on position 1 followed by a stretch of 4–15 pyrimidines. The first evidence suggested that TOR activates TOP mRNA translation through phosphorylation of S6K and RPS6 but this hypothesis was later questioned since TOP mRNA are normally translated in S6K-deficient mice (Meyuhas and Kahan, 2015). The Arabidopsis genome contains two tandem-repeated S6K genes and the proteins encoded by these genes are directly phosphorylated by TOR (Mahfouz et al., 2006; Schepetilnikov et al., 2011, 2013; Xiong et al., 2013). The phosphorylation level of S6K proteins is positively correlated to their capacity to phosphorylate RPS6 (Turck et al., 1998, 2004; Mahfouz et al., 2006; Browning and Bailey-Serres, 2015). In yeast, untargeted phosphoproteomic analyses pointed RPS6 to be the main phosphorylation target of TOR in the ribosome (Huber et al., 2009). In plants, RPS6 was found to be the major phosphorylated ribosomal protein in tomato cells and this phosphorylation was found to be reduced after heat stress (Scharf and Nover, 1982) or in oxygen deprived maize roots (Bailey-Serres and Freeling, 1990). Later a survey of post-translational modifications of the Arabidopsis ribosomal proteins only identified Ser240 in RPS6 and Ser137 in RPL13, together with acidic proteins, as being phosphorylated (Carroll et al., 2008). Multiple phosphorylation sites were detected in the RPS6 C-terminal region including Ser238 and Ser241 for maize (Williams et al., 2003) and Ser237 and Ser240 for Arabidopsis (Chang et al., 2005). Moreover Ser240 phosphorylation was found to be induced by light and high CO_2_ conditions (Turkina et al., 2011; Boex-Fontvieille et al., 2013). More recently, Nukarinen et al. (2016) have observed a strong induction of RPS6 Ser240 phosphorylation when the activity of the Sucrose non-fermenting 1-Related Kinase 1 (SnRK1) is decreased. However, despite the accumulation of data showing variations in plant RPS6 phosphorylation in response to several stresses, the precise role of this C-terminal phosphorylation in the regulation of translation remains largely elusive.

Since TOR was found to affect translation in plants, we undertook a global phosphoproteomic, transcriptomic and translatomic analysis of the ribosomal fraction after TOR inactivation. Interestingly, we observed a strong effect of TOR inactivation on the expression of nuclear-encoded plastidic ribosomal proteins (pRPs) and the main phosphorylation site controlled by TOR activity was found to be Ser240 in the cytoplasmic ribosomal protein (cRP) RPS6. Finally, we made use of this specific phosphorylation site to design a robust Western-based method for quantifying TOR activity in plant extracts.

## Materials and Methods

### Plant Materials and Growth Conditions

Seeds of two independent ethanol-inducible TOR RNAi lines (5.2 and 6.3, described in Deprost et al., 2007) as well as an ethanol-inducible GUS overexpressing line (as a control) (Deprost et al., 2007) were grown *in vitro* under long day conditions (16 h light/8 h night) for 7 days on solid 1/5 Murashige and Skoog medium supplemented with sucrose 0.3% (w/v) at a constant temperature of 25°C and a light intensity of 75 μE.m^-2^.s^-1^. The plants were subsequently treated with ethanol vapor for either 3 or 10 days. Whole plantlets from two independent biological replicates of each condition were then harvested in the middle of the light period and directly snap frozen in liquid nitrogen, grinded and subjected immediately to the ribosome enrichment protocol.

### Ribosome Enrichment

Ribosomal subunits (40S and 60S), monoribosomes (80S) and polyribosomes were isolated from the plantlet powder according to Bailey-Serres and Freeling (1990) with minor modifications. Freshly harvested and grinded plantlets were homogenized at a final concentration of 10% (w/v) in the ice-cold extraction buffer (0.2 M Tris-HCl [pH 9], 0.4 M KCl, 0.025 M EGTA, 0.035 M MgCl_2_, 0.2 M sucrose) supplemented with 2% (v/v) Triton X-100, 2% (v/v) Tween 20, 2% (v/v) NP-40 and 1% (w/v) sodium deoxycholate. The extracts were incubated on ice for 10 min to solubilize membrane-bound ribosomes and centrifuged at 2880 × *g* for 15 min at 4°C. The supernatants were layered over a sucrose cushion (0.04 M Tris-HCl [pH 9], 0.2 M KCl, 0.005 M EGTA, 0.03 M MgCl_2_, 1.75 M sucrose) and ultracentrifuged at 225 000 × *g* for 14 h. The ribosome enriched pellet was resuspended in 300 μl of Laemmli buffer (Laemmli, 1970) and denatured at 100°C for 10 min.

### LC-MS/MS Analysis

For the proteomic characterization, ribosome enriched fractions were first submitted to a short migration through the stacking gel of a SDS-PAGE, in order to remove the rRNA and the possible chemical contaminant, including detergents. After a Coomassie staining, the unique band of proteins, for each sample, was cut and divided into five pieces that were submitted, in gel, to the tryptic digestion, reduction and alkylation. Peptide containing fractions were then analyzed by nano LC-MS/MS as previously described (Boex-Fontvieille et al., 2013). Briefly on-line liquid chromatography was performed on a NanoLC-Ultra system (Eksigent). Eluted peptides were analyzed with a Q-Exactive mass spectrometer (Thermo Electron) using a nano-electrospray interface (non-coated capillary probe, 10 μ i.d; New Objective). Peptides and the corresponding proteins were identified and grouped with X!TandemPipeline using the X!Tandem Piledriver (2015.04.01) release (Craig and Beavis, 2004) and the TAIR10 protein library with the phosphorylation of serine, threonine and tyrosine as a potential peptide modification. Precursor mass tolerance was 10 ppm and fragment mass tolerance was 0.02 Th. Identified proteins were filtered and grouped using the X!TandemPipeline v3.3.4^1^. Data filtering was achieved according to a peptide *E*-value lower than 0.01. The false discovery rate (FDR) was estimated to 0.92%. Relative quantification was performed using the MassChroQ software (Valot et al., 2011) by peak area integration on extracted ion chromatograms (XICs) within a 10 ppm window, after LC-MS/MS chromatogram alignment and spike filtering.

### Phosphopeptide Enrichment

Arabidopsis seedlings grown on MS agar plates in standard 16/8 h and 21/17°C day/night conditions were transferred to liquid MS media supplemented with 10 μM NAA (Sigma-Aldrich). Total protein extracts were precipitated with 0.1 M ammonium acetate in 100% methanol, reduced, alkylated and digested overnight with trypsin (Promega, Madison, WI, USA) in 50 mM ammonium bicarbonate. Resulting peptides were vacuum-dried and re-suspended in 250 mM acetic acid with 30% acetonitrile for phosphopeptide enrichment with Phos-Select Iron Affinity Gel (Sigma-Aldrich) according to the protocol from Thingholm et al. (2008). Eluted phosphopeptides were desalted and analyzed by nano LC-MS/MS on a TripleTOF 5600 (Sciex, Canada) coupled a NanoLC-2DPlus system with nanoFlex ChiP module (Eksigent, Sciex).

### Transcriptome and Translatome Analysis

Transcriptomic and translatomic analyses were performed on two biological replicates using 7-day-old plantlets from the two independent TOR RNAi and GUS control lines grown *in vitro* and treated with ethanol for 24 h. Transcriptome analyses using CATMA arrays were performed on total RNA preparations as previously described (Moreau et al., 2012). For translatomic analyses total RNA was extracted and polysomal fractions were purified on sucrose gradients after ultracentrifugation as previously described (Deprost et al., 2007; Sormani et al., 2011). Polysome-bound RNAs were extracted using guanidinium hydroxychloride and precipitated by isopropanol and linear acrylamide as a carrier. Subsequently, RNAs were reverse transcribed and hybridized on CATMA arrays as described above for the determination of differentially translated mRNAs (Sormani et al., 2011). Statistical analysis of each comparison was based on two dye swaps and followed by the analysis described by Gagnot et al. (2008) and Moreau et al. (2012). Briefly, an array-by-array normalization was performed to remove systematic biases. To determine differentially expressed genes, we performed a paired *t*-test on the log ratios averaged on the dye swap. The raw *p*-values were adjusted by the Bonferroni method, which controls the family-wise error rate to keep a strong control of the false positives in a multiple-comparison context. We considered as being differentially expressed the probes with a Bonferroni *P*-value ≤0.05, as described by Gagnot et al. (2008). The results are available online in the CatDB database^2^.

### Antibody Production

Antibodies directed against phosphorylated RPS6A were obtained by conjugating to keyhole limpet hemocyanin the SRLpSSAAAKPSVTA (phosphoSer240) peptide (produced by Proteogenix, Schiltigheim, France) and injecting two New Zealand White female rabbits (performed by Proteogenix). Seven injections were performed over a period of 56 days. Then a preliminary ELISA test was performed at day 63 to evaluate the titer of the antibodies and the rabbits were bled at day 70. The obtained antisera were first depleted against immobilized non-phosphorylated peptide then specific antibodies were purified using the phosphorylated SRLpSSAAAKPSVTA peptide. The specificity of the purified antibodies was evaluated using an ELISA test with the phosphorylated and non-phosphorylated peptides (produced by Proteogenix) (Supplementary Figure S3).

### SDS-PAGE and Western Blotting

Primary antibodies used in this study are directed against mammalian RPS6 (Cell Signaling Technology #2317S), rapeseed RPL13 (Sáez-Vásquez et al., 2000) and Arabidopsis RPS14 (Agrisera AS09 477).

For detection of phosphorylated RPS6, total proteins were extracted from either wild-type (Col-0), TOR RNAi or control Arabidopsis lines using the Laemmli buffer and blotted with our RPS6 phospho-specific antibody. Bradford assay (Bio-Rad) was performed to quantify total protein concentrations. Ten micrograms of proteins were separated by SDS-PAGE gels and transferred to polyvinylidene difluoride membranes (PVDF, Bio-Rad) by electroblotting. Membranes were probed with either antiphospho-RPS6 (P-RPS6) rabbit polyclonal antiserum (dilution 1:5000) or with anti-RPS6 mouse monoclonal IgG (dilution 1:1000). Goat anti-rabbit IgG-HRP (horseradish peroxidase 1:2000, Santa Cruz Biotechnologies) and Goat anti-mouse IgG-HRP (1:2000, Santa Cruz Biotechnologies) were used as secondary antibodies. Immunodetection was performed by using enhanced chemiluminescent (ECL) substrates for HRP as recommended by the manufacturer (Clarity Western ECL blotting substrate Bio-Rad). Transferred proteins on PVDF membranes were visualized by Ponceau S staining.

### Motif Analysis

The 5′ UTR sequences of the ribosomal proteins mRNAs were obtained from the TAIR10 database^3^. The identification of the motifs was performed with the online MEME software^4^ (Bailey et al., 2009) with a motif recognition size comprised between 6 and 50 nt. Only the representative isoforms of the genes were used.

### Accession Numbers

Arabidopsis RPS6A: At4g31700 and RPS6B: At5g10360. Proteomic raw data are available in the Protic database under the following accession name: tor_inactivation^5^.

## Results

### Ribosome Enrichment by Density Ultracentrifugation

The aim of this study was to identify, by an untargeted proteomic analysis, modifications in the Arabidopsis ribosome fraction in response to TOR inactivation. First, we evaluated the suitability of our ribosome extraction method for LC-MS/MS analysis. To do so, 7-day-old seedlings of Arabidopsis (Col-0) were harvested and, to prevent protease, phosphatase, or kinase activities, immediately submitted to the ribosome purification protocol (see, Materials and Methods). Finally, high molecular weight particles, including polysomes, were pelleted by ultracentrifugation through a sucrose cushion.

**FIGURE 1:**
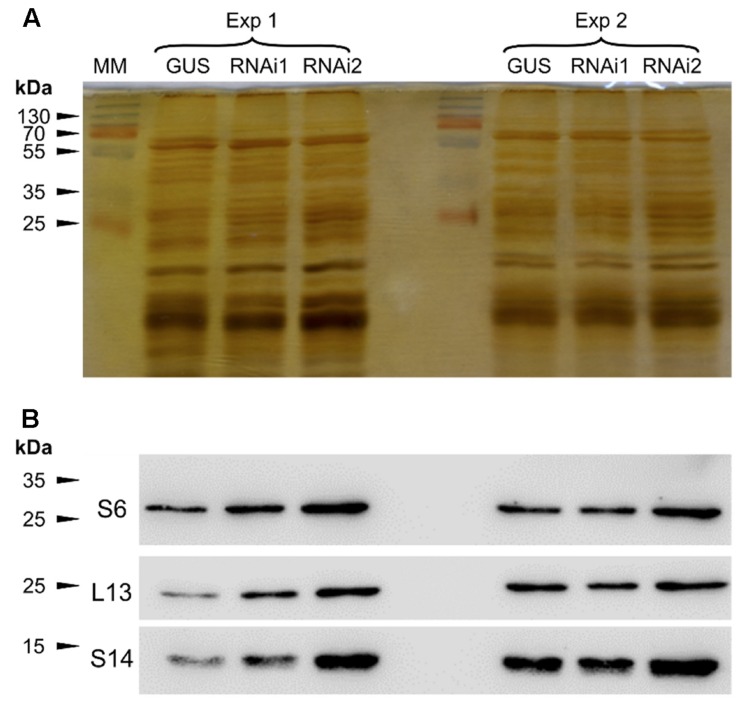
**Determination of ribosomal protein amounts prior to LC-MS/MS analyses.** Plant extracts were submitted to ultracentrifugation through a sucrose cushion to obtain a ribosome-enriched fraction. Pellets were resuspended in Laemmli buffer and analyzed for the abundance of ribosomal proteins by SDS-PAGE and Western blot. **(A)** Silver nitrate stained gel after SDS-PAGE. **(B)** Western blot against the RPS6, RPL13, and RPS14 ribosomal proteins. Exp1 and Exp2 correspond to two independent biological replicates. GUS is the control line, RNAi1 and RNAi2 are the two independent TOR RNAi lines. All the lines were induced with ethanol. MM, molecular marker.

We then submitted the ultracentrifugated fraction to SDS-PAGE and resolved proteins were stained using silver nitrate (**Figure 1A**). The obtained protein profile is typical of purified plant ribosomal fractions (Carroll et al., 2008). The presence of ribosomal proteins in this fraction was confirmed by Western blot analysis which allowed to normalize the protein fractions by diluting the samples according to Western blot quantifications (**Figure 1B**).

To inactivate TOR, we used two independent ethanol-inducible TOR RNAi lines (based on the AlcR/AlcA operon) that we previously obtained and characterized (Deprost et al., 2007) and compared them to a control line expressing an ethanol-inducible GUS gene (GUS control, Deprost et al., 2007; Dobrenel et al., 2011).

### Identification of Ribosomal Proteins by LC-MS/MS Analysis

**FIGURE 2:**
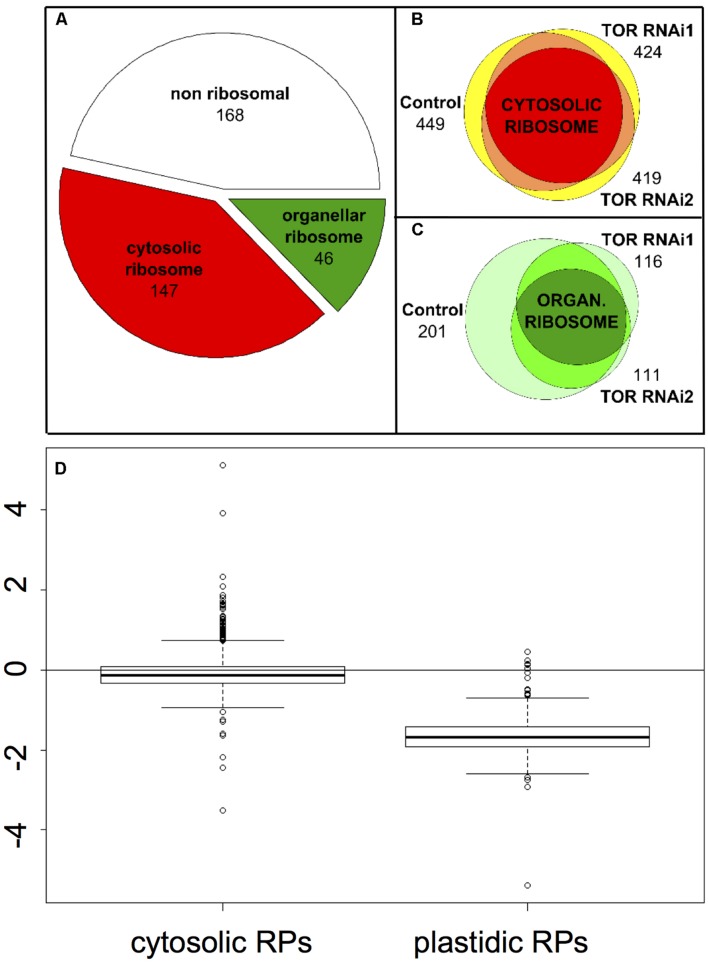
**Effect of TOR inactivation on the cytosol and organelle ribosomal proteins. (A)** Distribution of the proteins identified in at least one of the samples depending on whether they are part of the cytosolic ribosome, the organelle ribosome or non-ribosomal. **(B)** Venn diagram showing the number of peptides corresponding to the cytosolic ribosomal proteins identified in the GUS control and in the TOR RNAi lines. The intersections of two peptide sets are shown in orange and the intersection of the three sets in red. The total numbers of peptides are shown and the areas are representative of the number of common or specific peptides. **(C)**, same as **(B)** for the organelle ribosome except that intersections are shown in medium and dark green for two or three peptides sets, respectively. **(D)** Boxplot representing the relative abundance (log_2_ fold change) of the peptides derived from ribosomal proteins in the TOR RNAi compared to the GUS control lines depending of their localization in the cytosolic or plastidic ribosomes. For each peptide a mean abundance was obtained from the four repetitions (two independent experiments using the two RNAi lines) and the abundance ratios were compared.

Seedlings of two independent RNAi lines and of the GUS control were grown for 7 days *in vitro* and then treated with ethanol to induce TOR inactivation. We repeated the same experiment twice and then analyzed the six samples together. In order to remove eventual contaminating rRNA as well as the chemicals, the samples were first submitted to a short migration through a SDS-PAGE stacking gel. Proteomic as well as phosphoproteomic analyses by LC-MS/MS identified a total of 5936 spectra, corresponding to 1508 unique peptide sequences (raw data are available in the Protic database). Peptides were matched to protein sequences from TAIR10 and grouped according to sequence homology using the X!TandemPipeline with the phosphorylation of serine, threonine and tyrosine as a potential peptide modification. By this method, 361 different proteins were potentially identified by at least two peptide sequences, belonging to 217 groups (corresponding presumably to protein families, based on sequence homologies). Among these 361 proteins, 210 were identified by the presence of at least two proteotypic (i.e., specific of a given protein) peptides. Based on the previous annotations of the ribosomal proteins (Sormani et al., 2011; Tiller and Bock, 2014; Hummel et al., 2015), we found that more than half of the 361 proteins were ribosomal proteins (147 correspond to cytosolic ribosomes and 46 to organelle ribosomes) (**Figure 2A**). Using an *in silico* analysis, Sormani et al. (2011) refined the annotation of ribosomal proteins (including plastidic and mitochondrial ones). We used this list to identify the proteins (and therefore the peptides) corresponding to the mitochondrial and plastidic ribosomes. All identified organellar peptides corresponded to plastidic ribosomes.

Target of Rapamycin inactivation in the RNAi lines did not largely affect the number of detected peptides originating from the cytosolic ribosomes and thus most peptides were found both in the RNAi and in the control lines (between 419 and 449 peptides for RNAi2 and GUS lines, respectively; **Figure 2B**). Conversely the pool of peptides coming from pRPs was specifically depleted in the TOR RNAi lines with only 116 and 111 peptides detected for the TOR RNAi1 and RNAi2 lines, respectively, compared to 201 peptides in the control GUS line (**Figure 2C**). One third of the pRPs peptides were only present in the control GUS line (74 out of 218) whereas a much smaller number of peptides (17) were specifically found in the RNAi lines.

### Quantitative Analysis of the Expression of Ribosomal Protein Encoding Genes

In order to exclude biases that could be caused by potential technical issues, like the mass spectrometer being occupied by some abundant peptides eluting near the peptides of interest, we quantified the traces of the peaks corresponding to the identified peptides of the plastidic and cytoplasmic RPs. These peak areas were then compared between the RNAi and GUS control lines for each peptide (**Figure 2D**). Such a quantitative approach confirmed that most of peptides resulting from the fragmentation of the pRPs are indeed less abundant in the TOR RNAi samples. This also confirmed that TOR inactivation did not have any strong effect on the accumulation of the cRPs peptides (**Figure 2D**). Thus these results suggest a global decrease in the abundance of pRPs resulting in a lower number and amount of peptides detected in the LC-MS/MS analysis.

**FIGURE 3:**
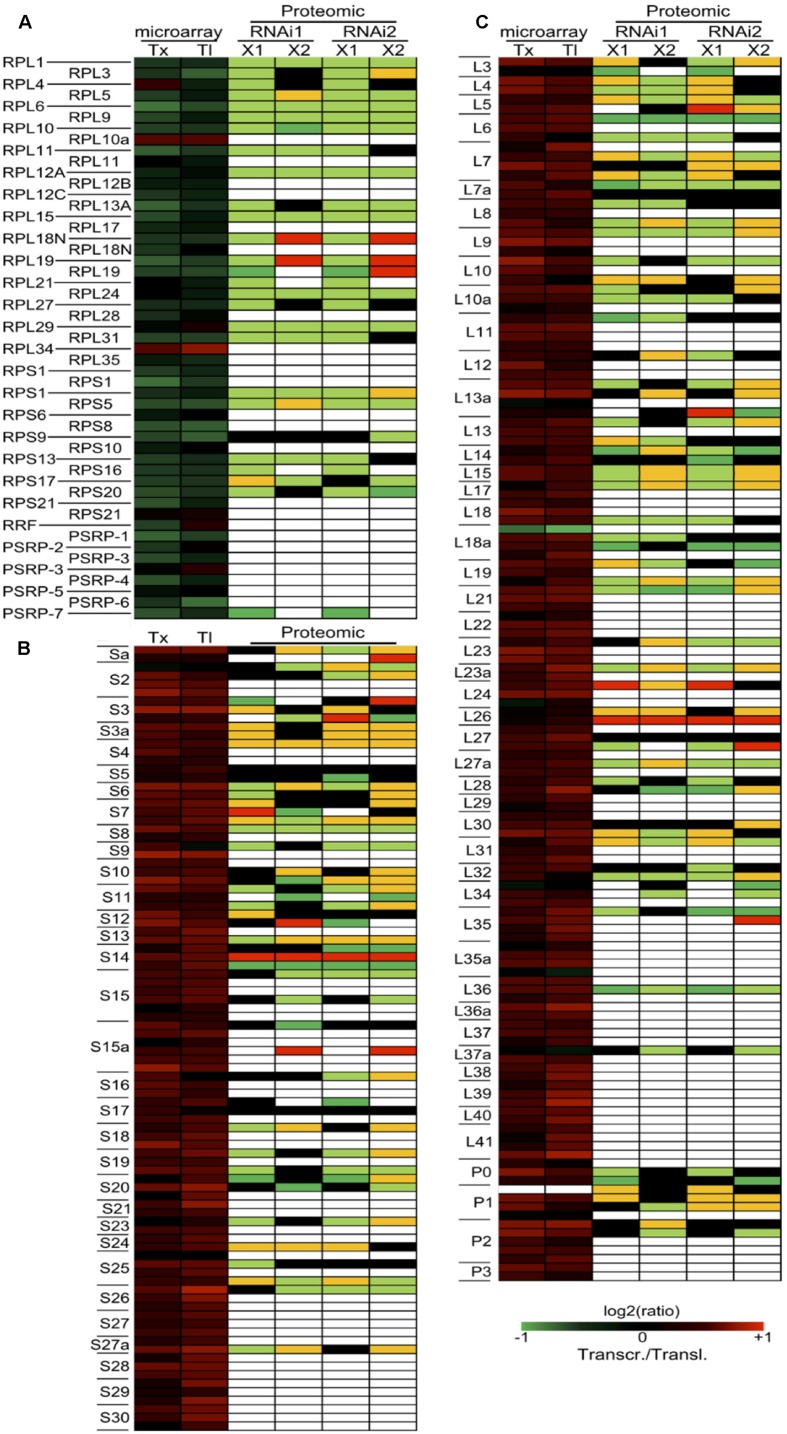
**Effect of TOR inactivation on the transcription, translation, and abundance of ribosomal proteins. (A)** Expression of the nuclear genes coding for chloroplastic ribosomal proteins at the total mRNA level (transcriptomic analysis: Tx), at the polysome-bound mRNA level (translatomic analysis: Tl) and at the protein level (proteomic analysis). **(B,C)** Expression level of the genes coding for the cytosolic ribosomal proteins and localized in the small or large ribosome subunit, respectively. For the transcriptome and translatome experiments, mRNA abundances in the TOR RNAi lines were compared to the GUS control line treated with the same 24 h ethanol induction time. The results represent the mean of the two TOR RNAi lines in the two independent biological replicates. Intensity ratios are shown in log_2_ scale according to the color scale shown in the figure on the bottom right for transcriptomic and translatomic experiments (Transcr./Transl.). For the proteomic experiment, two independent biological replicates (X1 and X2) using the two TOR RNAi lines are presented. Orange and pale green represent a quantitative up- and down-regulation, respectively, and red and dark green represent a qualitative regulation, respectively (peptides either present or absent). Only the proteotypic spectra were used. Black boxes indicate no change and white boxes represent missing data.

We then used the number of spectra (or peptide hits) per protein to estimate the protein abundance for the pRPs and cRPs. Indeed it has been shown that there is a proportional relationship between these two values (Allet et al., 2004; Liu et al., 2004). We exclusively used the spectra corresponding to proteotypic peptides in order to avoid a bias that would be caused by the very high sequence homology within ribosomal protein families. By this method, we showed a coordinated down-regulation of the pRPs while the cRPs have a much less coordinated profile and are globally only slightly affected by the TOR inactivation (**Figure 3**). To better understand the role of TOR in regulating plant gene expression, we performed transcriptomic and translatomic analyses after TOR inactivation, in which we monitored the mRNA levels on total and polysome-bound RNA samples. The variations in the abundance of polysome-bound mRNA were determined after purification of polysomes on a sucrose gradient, extraction of RNA and microarray hybridization. To better identify the primary TOR-regulated mRNA targets, we decided to shorten the time of ethanol-mediated RNAi induction. Two biological repetitions were performed using each time the two independent RNAi lines. The resulting four transcriptomic and translatomic experiments were submitted to a statistical analysis to identify common differentially expressed genes when compared to the GUS control lines (see Materials and Methods). When focusing on the ribosomal proteins, we found a large difference in the expression profiles of the corresponding nuclear genes depending on whether the gene product is part of the cytoplasmic or the plastidic ribosome (**Figure 3**; Supplementary Table S1). First, almost all detected nuclear-encoded mRNA coding for pRPs, whether located in the small or in the large subunit, showed either no change in abundance or were down-regulated after TOR inactivation when compared to the GUS control line treated with ethanol (**Figure 3A**). On the opposite mRNAs coding for cRPs were mostly up-regulated (**Figures 3B,C**) except for RPL18a-1 which was the only transcript showing a reproducible decrease in abundance. The translatomic analysis mostly mirrored the transcriptomic variations but for some genes coding for cytosolic ribosomes, like RPL40 and RPL41, the total mRNA abundance did not vary significantly whereas they were more engaged in polysomes compared to the GUS control, suggesting a higher translation of these genes (**Figure 3C**). Taken together, these data suggest that there is a coordinated down regulation of the nuclear genes coding for the pRPs at the transcriptional, translational (polysomal loading), and protein levels in response to TOR inactivation (**Figure 3A**).

**FIGURE 4:**
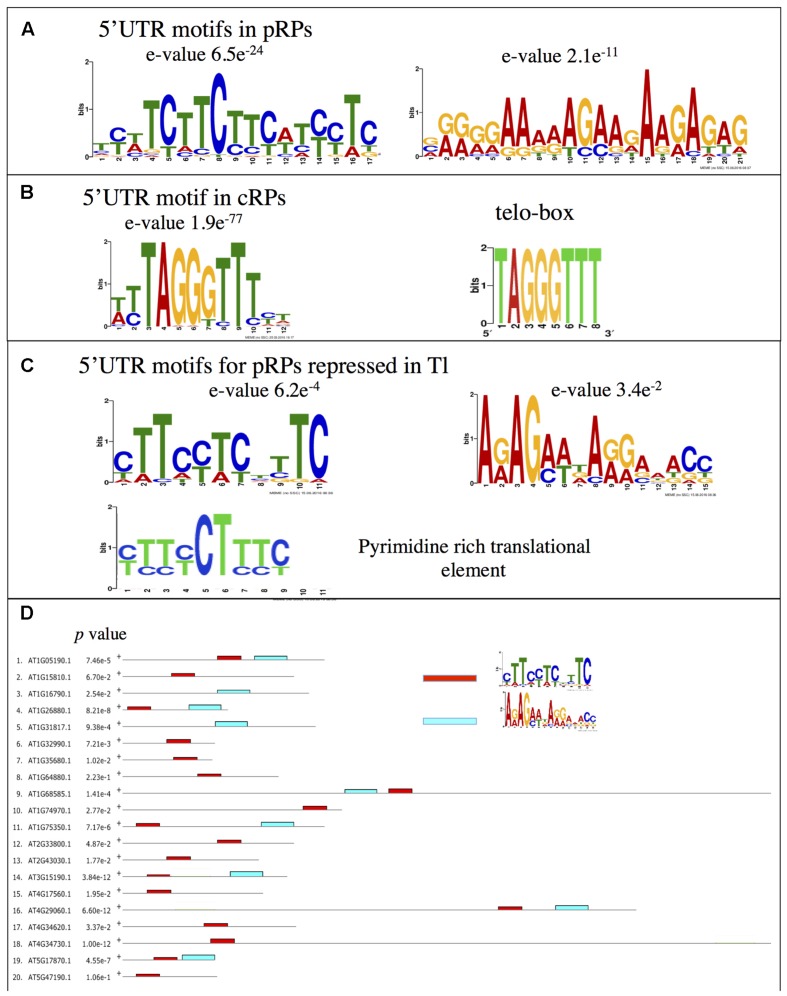
**Motifs identified in the 5′ UTRs of genes coding for ribosomal proteins. (A)** Motifs identified as being significantly enriched in the 5′ UTRs of the nuclear genes coding for the plastidic RPs after analysis by the MEME software. **(B)** Same for genes coding for the cytosolic RPs. The telo-box motif found in cRPs promoters is shown. **(C)** Same as for **(A)** but only the 5′ UTRs of the genes showing a down-regulation in the translatome experiments following TOR inactivation were kept. **(D)** Positions of the motifs identified in **(C)** in the 5′ UTRs of the analyzed sequences. See Supplementary Figure S1 for details.

Next we examined whether this co-regulation involves the recognition of a conserved motif in the 5′ UTR sequences of the nuclear-encoded mRNA coding for pRPs. These sequences were analyzed for enriched motifs using the MEME software (Bailey and Elkan, 1994). A strongly significant motif composed of a stretch of pyrimidines was identified in this set of 5′ UTRs (**Figure 4A**). This sequence is reminiscent of the animal TOP motif found within the 5′UTR of mammalian ribosomal protein encoding mRNAs, which confers translational regulation by TOR (Meyuhas and Kahan, 2015). The MEME analysis also revealed the presence of a second A/G-enriched motif (**Figure 4A**). On the contrary, the 5′ UTRs of the mRNAs coding for cRPs were significantly enriched for a TTTAGGGTTT motif (**Figure 4B**), which is similar to the telo-box consensus sequence already identified in the promoters of these genes (**Figure 4B**) (McIntosh et al., 2011; Wang et al., 2011). Next we analyzed the 5′ UTRs of the nuclear genes coding for pRPs, which were less engaged in polysomes after TOR inactivation (**Figure 3A**). MEME analysis identified a shorter pyrimidine-rich motif that is more similar to canonical TOP motifs, but even more to the pyrimidine-rich translational element (PRTE), a motif identified in animal genes which translation is controlled by TOR (**Figure 4C**) (Hsieh et al., 2012). This motif is found in the majority of animal TOR targets and, unlike conventional TOP motifs, does not reside at the start of the mRNA sequence (Hsieh et al., 2012). Consistently, the motif we identified is also rarely present at the start of the analyzed mRNAs (**Figure 4D**; Supplementary Figure S1) but is significantly enriched in pRP transcripts translation of which is affected by TOR inactivation. A purine-rich motif was also identified in this subset of genes and was often found 3′ to the PRTE-like motif (**Figure 4D**).

We then mined the public Genevestigator transcriptome database (Hruz et al., 2008) for information about the expression profile of plastidic ribosomal genes encoded by the nuclear genome. Interestingly these genes were found to be down-regulated in estradiol-inducible TOR RNAi lines (Xiong et al., 2013; Supplementary Figure S2) which suggests that TOR inactivation reproducibly reduces their expression. Nuclear genes coding for pRPs are also strongly induced during germination or after light treatment, which is consistent with their role in the formation of the photosynthetic machinery. Conversely they were repressed in response to various stresses such as pathogen infection, drought, hypoxia, or increased temperature as well as in response to extended night. Finally these genes were slightly induced in one experiment of sucrose feeding but globally repressed in several experiments of nitrogen starvation (Supplementary Figure S2).

### TOR Dependent Phosphorylation of RPS6 on Ser240

**FIGURE 5:**
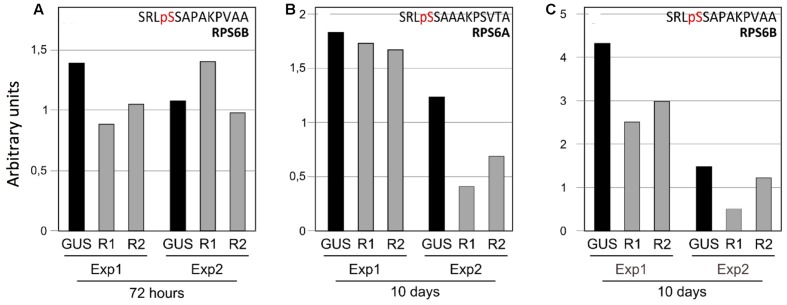
**Downregulation of RPS6A and RPS6B phosphorylation in response to TOR inactivation. (A)** Phosphorylation level of the RPS6B C-terminal peptide after 72 h of ethanol treatment. **(B)** Same for the RPS6A and **(C)** for the RPS6B proteins after 10 days of ethanol treatment. Each bar corresponds to the peak intensity of the phosphorylated peptide normalized by a proteotypic peptide from the same protein (GENDLPGLTDTEKPR for RPS6A and GVSDLPGLTDTEKPR for RPS6B). For each experiment, the results of the GUS control (GUS), the TOR RNAi1 (R1) and the TOR RNAi2 (R2) lines are presented. Exp1 and Exp2 correspond to two independent biological replicates with 72 h or 10 days of TOR inactivation.

**FIGURE 6:**
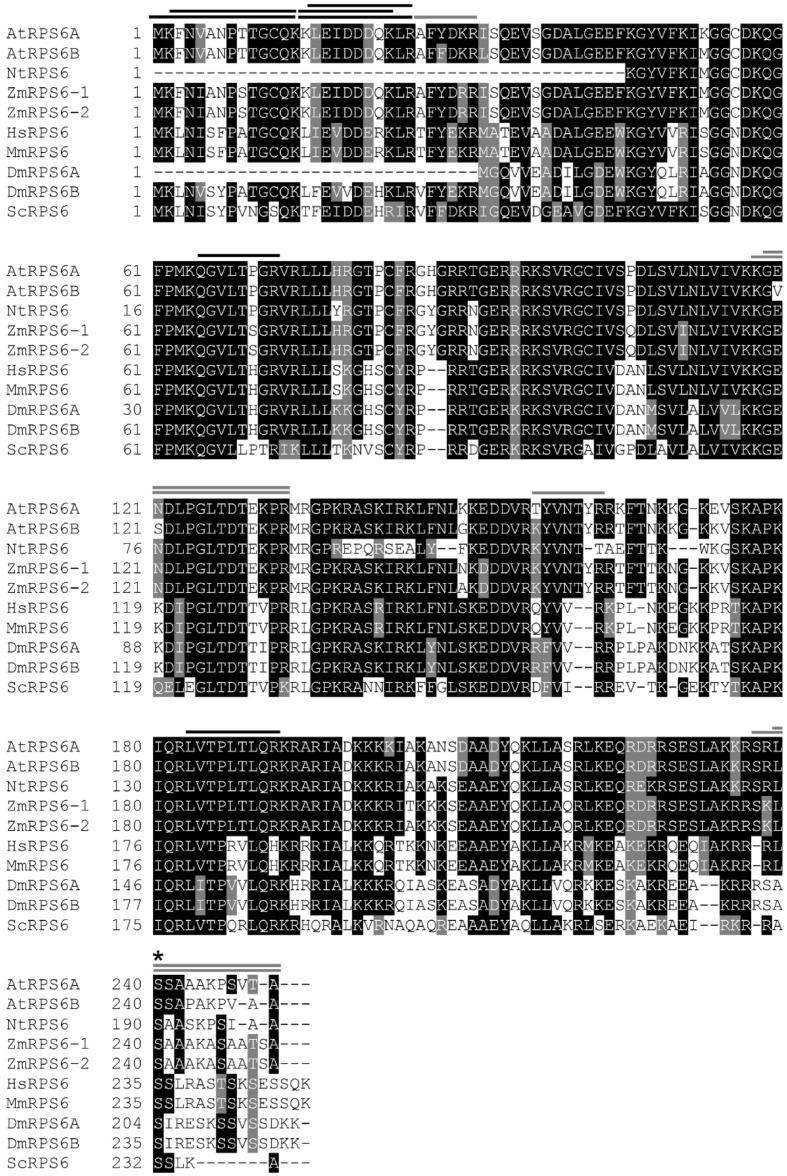
**Comparison of RPS6 amino acid sequences.** Arabidopsis RPS6s (AtRPS6A, At4g31700.1, and AtRPS6B, At5g10360.1), *Nicotiana tabacum* RPS6 (NtRPS6, P29345.2), *Zea mays* RPS6s (ZmRPS6-1, NP_001105544.1 and ZmRPS6-2, NP_001105634.1), *Homo sapiens* RPS6 (HsRPS6, NP_001001.2), *Mus musculus* RPS6 (MmRPS6, NP_033122.1), *Drosophila melanogaster* RPS6s (DmRPS6A, NP_727213.1 and DmRPS6B, NP_511073.1) and *Saccharomyces cerevisiae* RPS6 (ScRPS6, NP_015235.1) protein sequences were aligned by T-Coffee (http://tcoffee.crg.cat). The level of conservation is represented by a color code in which the most conserved residues are in black, those with intermediate conservation are gray and the least conserved are in white. RPS6 peptides identified in Arabidopsis by the proteomic analysis are represented by lines over the corresponding amino acid sequences (black for the peptides common to the two isoforms and gray for peptides specific to one isoform). An asterisk shows the conserved phosphorylated Ser240 residue.

Even if most of the cRPs were not affected by TOR inactivation (**Figure 2D**) we found 30 peptides which were significantly down regulated (*t*-test *p*-value < 0.05; Supplementary Table S2). In most of the case, these peptides are not proteotypic, thus making the conclusions more complicated. The two cytosolic RPS6 paralogs were identified with three different proteotypic peptides that are significantly down-regulated in a quantitative manner following TOR inactivation (**Figure 3B**). In total, 15 peptides were detected for this protein family. Among these 15 peptides, we identified a phosphorylated peptide corresponding to the RPS6B C-terminal extremity. The X!Tandem analysis predicted a phosphorylation site on Ser240 (SRLpSSAPAKPVAA: **Figure 5**; Supplementary Figure S3). This serine seems to be conserved in the eukaryotic RPS6 proteins, including plants, and was previously identified as being phosphorylated following TOR activation in yeast and animals (Pende, 2006; Meyuhas, 2008; Yerlikaya et al., 2016) (**Figure 6**). In order to clarify the actual position of the phosphorylated residue in this peptide, the phosphoproteomic results were submitted to a Phoscalc statistical analysis (MacLean et al., 2008). This analysis failed to clearly identify the phosphorylated site between Ser237, 240 or 241 (Supplementary Figures S3C,D). Nevertheless it showed that this RPS6B C-terminal peptide carries only one phosphate group. An independent phosphoproteomic analysis of Arabidopsis seedlings treated with auxin was also performed (see Materials and Methods). Indeed previous studies suggested that auxin mediates TOR, and thus S6K1, activation in Arabidopsis as shown by phosphorylation of TOR at Ser2424 and S6K1 at Thr449 (Schepetilnikov et al., 2013). Since S6K1 is directly involved in the phosphorylation of RPS6, we asked whether this ribosomal protein is phosphorylated at Ser240 in Arabidopsis extracts treated by auxin. After purification of the phosphopeptides by immobilized metal affinity chromatography (IMAC), Ser240 was again identified as a potential phosphorylation site both in RPS6A and B (Supplementary Table S3; Supplementary Figures S3E,F). In some cases RPS6A Ser240 was identified together with Ser237 (pSRLpSSAPAKPVAA) but again the discrimination between phosphorylated Ser240 and Ser241 was not always possible (Supplementary Table S3).

Even if the precise localization of the phosphorylated serine residue in the RPS6B protein C-terminus could not be determined without ambiguities, we compared the abundance of the C-terminal monophospho-SRLSSAPAKPVAA peptide between the TOR RNAi lines and the GUS controls. After 72 h of exposure to ethanol we observed a modest decrease in the RPS6B C-terminal phosphorylated peptide (**Figure 5A**). This decrease in phosphorylation was only observed in one of two experiments. Since the abundance of the phosphorylated peptide was already low in the control line for the second experiment, it could be that the decrease in phosphorylation level was less obvious in this case. The corresponding RPS6A peptide was not found in this analysis. Thus to confirm this TOR-dependent decrease in RPS6 C-terminus phosphorylation, we performed a longer silencing induction by ethanol for 10 days. As for the 72 h treatment, biological duplicates have been used. The abundance of the monophospho C-terminal peptides was compared for both RPS6A and RPS6B proteins between the RNAi and the control GUS lines (**Figures 5B,C**). The amount of phosphorylated C-terminal peptide was lower (with a mean of 40% decrease) for both the RPS6A and the RPS6B proteins in the TOR RNAi lines when treated with ethanol for 10 days.

**FIGURE 7:**
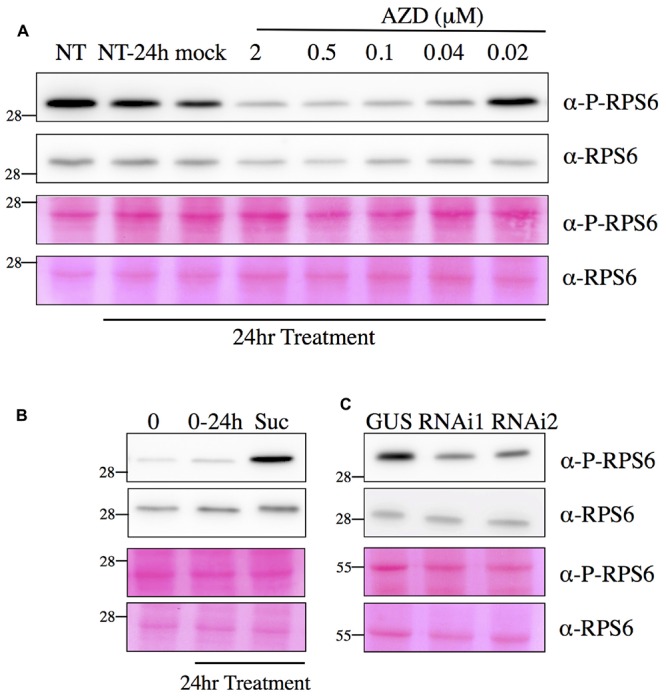
**Detection of RPS6 phosphorylation by Western blot assay.** Total protein extracts obtained from seedlings were separated by SDS-PAGE and blotted onto a membrane. After incubation with the phospho-specific antibody against RPS6 Ser240 (P-RPS6) or a monoclonal antibody against total mammalian RPS6, blots were revealed by a secondary antibody linked to HRP activity and imaged with a CCD camera (see Materials and Methods for details and Supplementary Figure S4). **(A)** AZD treatments inhibit Ser240 RPS6 phosphorylation. Six day-old seedlings were either mock or AZD treated for 24 h. NT, non-treated plants at time 0 and 24 h. **(B)** Sucrose treatment induces RPS6 phosphorylation. Six day-old seedlings were transferred to sugar-free medium for 24 h and then either mock (0–24 h) or sucrose (0,5%) treated for 24 h. 0: plants before sucrose induction at time 0. **(C)** Silencing of TOR decreases RPS6 phosphorylation. The control (GUS) or TOR RNAi lines were grown for 7 days and induced with 5% (v/v) ethanol (EtOH). Bottom panels show Ponceau Red staining of the membranes.

To confirm this phosphoproteomic analysis, a phospho-specific antibody was raised against a synthetic SRLpSSAAAKPSVTA peptide in which only Ser240 was phosphorylated. The obtained polyclonal antibody was purified against this peptide and the eluted antibody fraction detected a single band corresponding in size to RPS6 proteins (Supplementary Figure S4A; **Figure 7**). Furthermore this antibody was found to be highly specific for the phosphorylated SRLpSSAAAKPSVTA peptide, showing no reaction with the control non-phosphorylated peptide in an ELISA test (Supplementary Figure S4B).

**FIGURE 8:**
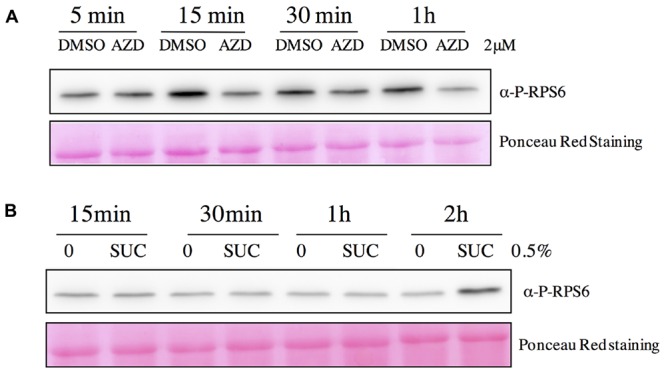
**Kinetics of variations in RPS6 phosphorylation following AZD-8055 or sucrose addition.** Total protein extracts obtained from seedlings were separated by SDS-PAGE and blotted onto a membrane. After incubation with the phospho-specific antibody against RPS6 Ser240 (P-RPS6) blots were revealed by a secondary antibody linked to HRP activity and imaged with a CCD camera. **(A)** Kinetics of the inhibition of RPS6 Ser240 phosphorylation by AZD 8055. Seven day-old seedlings were either mock (DMSO) or AZD-8055 (2 μM) treated and then harvested at the indicated time. **(B)** Kinetics of the induction of RPS6 Ser240 phosphorylation by sucrose. Seven day-old seedlings were transferred in sugar-free medium for 24 h and then either mock (0) or sucrose (0.5%) treated. Seedlings were harvested at the indicated time. Bottom panels show Ponceau Red staining of the membranes.

This antibody was used in an optimized Western blot assay and the obtained signal was very strong even when the antibody was diluted 1/5000. This band co-migrated with the band decorated by a specific monoclonal antibody against mammalian RPS6 (**Figure 7**) which was subsequently used to quantify the amount of total RPS6 in protein extracts. Both treating Arabidopsis seedlings with AZD-8055, a strong and specific second generation TOR inhibitor (Montané and Menand, 2013), or silencing the expression of TOR by a 7-day ethanol treatment, resulted in a significant and dose-dependent decrease in the signal obtained with the RPS6 phospho-specific antibody, whereas the total amount of RPS6 protein only decreased slightly (**Figures 7A,C**). Since this antibody is highly specific for the phosphorylated RPS6 protein, this confirms that there is a reproducible decrease in Ser240 phosphorylation level following TOR inactivation. Accordingly it was previously shown in animals that AZD inhibits TOR, and as a consequence S6K activity and RPS6 phosphorylation (Chresta et al., 2010). TOR and S6K were previously shown to be activated by sugars like sucrose and glucose (Xiong et al., 2013). Consistently RPS6 phosphorylation was augmented by the addition of sucrose when supplied to sugar-starved Arabidopsis seedlings (**Figure 7B**). Next we examined the kinetic of changes in RPS6 phosphorylation after either AZD-8055 or sucrose treatments (**Figure 8**). As soon as 1 h after AZD addition, a significant decrease in Ser240 phosphorylation was observed (**Figure 8A**). For sucrose, an increase in phosphorylation was detected 2 h after supply. However it is difficult at this stage to discriminate between a direct signaling effect of sucrose and an indirect consequence of sugar metabolism (**Figure 8B**). Altogether these data suggest a strong positive correlation between the RPS6 phosphorylation level, as detected by this specific polyclonal antibody, and TOR activity. Therefore, it seems that in plants, like in animals and yeast, RPS6 phosphorylation can be used as a robust and sensitive readout for TOR activity.

## Discussion

Several studies have investigated the impact of TOR inhibition in Arabidopsis on transcript or metabolite levels (Deprost et al., 2007; Moreau et al., 2012; Caldana et al., 2013; Xiong et al., 2013; Dong et al., 2015) but hitherto the global proteome has not been examined. In this paper, we investigated the expression of ribosomal proteins and genes using transcriptome, translatome, proteome and phosphoproteome analyses following silencing of the TOR gene. Concerning the cytoplasmic ribosome, we identified in our proteomic experiments 65 families of ribosomal proteins, corresponding to 69 ribosomal protein isoforms identified with at least two proteotypic peptides. Hummel et al. (2015) identified 165 cRPs isoforms by LC-MS/MS after a tryptic digestion. We were able to find more than one third of these protein paralogs and only 16 families were not found in this analysis. Seven of these 16 families cannot be identified by LC-MS after a tryptic digestion mainly because of the small size of the resulting peptides due to their high content in lysine and arginine. Thus, only nine out of the 81 ribosomal protein families were missing in our analysis (RPL22, RPL35a, RPL38, RPP3, RPS27, RPS28, RPS29, RPS30, and RACK1). Moreover, this work has been focusing solely on young seedlings while Hummel et al. (2015) were also using rosettes in their analysis and some specific paralogs may be expressed only in specific tissues or at some specific developmental stages (Weijers et al., 2001; Sormani et al., 2011). Since we performed our proteomic analyses using plants silenced for TOR expression by a long ethanol treatment (3 and 10 days), our analysis of changes in protein abundance may reveal steady-state long-term, and sometimes indirect, adaptation to a decrease in TOR activity.

In plastids translation occurs on 70S bacterial-type ribosomes. About half of the plastid small ribosome subunit (30S) proteins and most of the large subunit (50S) proteins are encoded by the nuclear genome (Yamaguchi and Subramanian, 2000). TOR silencing (Deprost et al., 2007; Xiong and Sheen, 2012; Caldana et al., 2013), inhibition by rapamycin (Sormani et al., 2007; Ren et al., 2011) or by AZD-8055 (Montané and Menand, 2013; Li et al., 2015) as well as mutations affecting the TORC1 complex (Moreau et al., 2012; Kravchenko et al., 2015) consistently result in leaf chlorosis and yellowing. We show here that TOR inhibition results in a coordinated decrease in pRP expression, at the level of protein abundance but also at the total and translated mRNA levels, which could explain these chlorotic phenotypes (**Figure 3**). Whether this is the result of a decreased synthesis or an increased degradation of the chloroplast components by autophagy, which is induced after TOR inactivation (Liu and Bassham, 2010), remains to be determined. Interestingly the expression of nuclear genes coding for cytosolic proteins was found to be mostly induced whereas the level of proteins often decreased. The same trends were observed in N-limited *Chlamydomonas* where the levels of pRPs as well as their corresponding mRNAs decreased in response to N starvation whereas only protein levels decreased for cRPs (Schmollinger et al., 2014). The same effect of TOR inactivation on the expression profile of the pRPs was confirmed by the comparison with transcriptomic data obtained using estradiol-inducible TOR RNAi line (Supplementary Figure S2) (Xiong et al., 2013). Interestingly, nuclear genes coding for pRPs were down-regulated in response to several abiotic or biotic stresses suggesting that they may play an important role in the adaptation to stresses (Supplementary Figure S2). These genes were also repressed by ABA but strongly induced by the application of brassinolide. This is consistent with the inhibitory effects of ABA on the growth-promoting hormones like brassinosteroid and with the role of TOR in brassinosteroid (Zhang et al., 2009, 2016) and ABA signaling (Kravchenko et al., 2015; Li et al., 2015), which could result in TOR inhibition. As reported earlier by Pal et al. (2013) we found diverse and uncoordinated responses to variations in sugar supply for nuclear genes coding for pRPS while their expression was repressed by nitrogen starvation as observed in Chlamydomonas (Schmollinger et al., 2014). Altogether, these data suggest that nuclear genes coding for the pRPs are controlled by TOR at multiple levels to integrate environmental cues for the regulation of chloroplastic translation.

In animals TOR is known to regulate the translation of TOP-containing mRNAs (Hsieh et al., 2012; Thoreen et al., 2012; Meyuhas and Kahan, 2015). This motif is particularly present in the 5′ UTR of genes coding for ribosomal proteins or components of the translation machinery (Meyuhas and Kahan, 2015). Using a MEME analysis we did not detect any specific enrichment for TOP motif in cRPs. The most abundant motif was related to the telo-box (**Figure 4A**). This DNA motif is found in the 5′ regions, often close to the start codon, of nuclear genes coding for both mitochondrial and cytosolic RPs, but not in the genes encoding pRPs (Wang et al., 2011). Conversely, we found a highly significant occurrence of a pyrimidine-rich motif in the 5′ UTR of nuclear genes coding for pRPs. This motif is reminiscent of TOP motifs (**Figure 4A**). A TOP-like motif was also previously identified in mRNA coding for ribosomal proteins in maize embryonic axes (Jiménez-López et al., 2011) but these motifs are so far poorly described in plants. Canonical TOP motifs are located at the start of the mRNA, which is not the case in our analysis. Nevertheless, a previous study has demonstrated the presence of several transcription start sites in genes coding for pRPs (Lagrange et al., 1993). Interestingly for the plastidic RPL21 gene, the start site specifically used in leaves produced a mRNA starting with a canonical TOP motif. The 5′ UTRs of cRP genes which are less translated after TOR inactivation were enriched in a motif that is strikingly similar to the PRTE motif found within the 5′ UTRs of animal genes controlled by TOR at the level of translation (**Figures 4C,D**) (Hsieh et al., 2012). It is thus tempting to hypothesize that TOR has been recruited in plants to regulate specifically in leaves the translation of nuclear mRNAs coding for chloroplastic ribosomal proteins. It was previously shown that translation of animal TOP-containing mRNAs can be differentially regulated *in vitro* in a wheat germ extract (Shama and Meyuhas, 1996) and that auxin stimulates S6 ribosomal protein phosphorylation on maize ribosomes and the recruitment of TOP-like mRNAs for translation (Beltrán-Peña et al., 2002). Since TOR is also activated by auxin (Schepetilnikov et al., 2013) these data suggest that plant mRNAs containing TOP-related motifs could also be regulated in a TOR- and phosphorylated S6-dependent manner.

Only phosphorylation of the RPS6 protein could be identified in a reproducible manner in previous unbiased phosphoproteomic analyses of the ribosomes performed in eukaryotes (Huber et al., 2009; Hsu et al., 2011; Yu et al., 2011). Nevertheless, despite a wealth of studies and several hypotheses, the precise biological role of these conserved phosphorylation events remains disputed and unclear. For example, expression of human RPS6 containing alanine at all phosphorylated serine residues did not modify the overall translation rate, even for TOP mRNAs (Ruvinsky et al., 2005). Instead cell growth and size as well as ribosome biogenesis were affected (Ruvinsky et al., 2005; Chauvin et al., 2014). The same result was observed in yeast expressing non-phoshorylatable RPS6 (Yerlikaya et al., 2016). However, phosphorylation of the C-terminal Ser residues of RPS6 has been used as a robust and recognized readout for TOR activity in animals and yeast (Meyuhas, 2008, 2015; Yerlikaya et al., 2016). In this study, a decrease in RPS6 phosphorylation in response to TOR inactivation was observed (**Figures 5** and **7**). The phosphoproteomic analysis identified a C-terminal phosphorylation site in both the RPS6A and RPS6B proteins that is TOR activity-dependent without unambiguously determining which of the C-terminal serine residues is modified. In Arabidopsis Ser237 was previously identified by MALDI-TOF as being phosphorylated but the absence of fragmentation in the C-terminal region hindered the precise localization of the other modification sites by MS/MS analysis (Chang et al., 2005). Several phosphoproteomic studies of the plant ribosome have already shown the presence of a phosphorylation site in the C-terminal peptide of the RPS6 and have suggested that the Ser240 could be one of the modified residues together with Ser229, 231, or 237 (Carroll et al., 2008; Turkina et al., 2011; Boex-Fontvieille et al., 2013). A global phosphoproteome analysis of Arabidopsis identified Ser237 and 240 as being phosphorylated together with Ser247 and Thr249 (Reiland et al., 2009). Ser240 is conserved in all plant RPS6 sequences whereas Ser241 is missing in the maize and tobacco sequences (**Figure 6**). Conversely Ser237 is found in all plant sequences but only Ser240 can be aligned with one of the known phosphorylated serine in the yeast (Ser232) or human (Ser235) RPS6 sequence (**Figure 6**; Meyuhas, 2015). It is well known in yeast and animals that TOR activity controls RPS6 phosphorylation through activation of S6K (Wullschleger et al., 2006; Biever et al., 2015; Meyuhas, 2015) and Mahfouz et al. (2006) have established that TOR interacts with S6K through RAPTOR to activate RPS6 phosphorylation. Nevertheless it should be noted that S6K is also activated by the 3-phosphoinositide-dependent protein kinase 1 (PDK1) which operated after S6K phosphorylation by TOR (Mahfouz et al., 2006; Otterhag et al., 2006). Interestingly the SnRK1 kinase, which probably acts antagonistically to TOR (Dobrenel et al., 2016), was recently shown to interact with and phosphorylate RAPTOR in Arabidopsis (Nukarinen et al., 2016). Moreover, a strong increase in RPS6 Ser240 phosphorylation was also observed after SnRK1 inactivation. This is coherent with the hypothesis that SnRK1 inhibits TOR activity, and hence RPS6 phosphorylation, presumably through RAPTOR phosphorylation (Nukarinen et al., 2016).

Western blot assays using the RPS6 Ser240 phospho-specific antibody demonstrated that phosphorylation of this residue decreased following TOR inactivation either by silencing or by using a specific inhibitor (**Figure 7**). Therefore this assay could be used as a TOR readout in plants. Previous assays for TOR activity in plants were based on the detection of Thr449 phosphorylation in S6K by commercial antibodies directed against phosphorylated Thr389 in animal S6K. However, these antibodies produce many non-specific bands in Western blot assays (Schepetilnikov et al., 2011; Xiong and Sheen, 2012). Moreover the abundance of plant S6K is low in plants whereas RPS6 is present in large amounts.

Taken together these data show that the TOR-dependent C-terminal RPS6 phosphorylation is conserved in plants like in other eukaryotes. We have taken advantage of this conserved phosphorylation to design a sensitive and specific assay to monitor TOR activity in plants. The question that remains open is the biological role of RPS6 phosphorylation. Structural studies have shown that RPS6 is accessible to the solvent, and hence to kinases, but the disordered C-terminal region is unfortunately absent from the resolved ribosome structure (Khatter et al., 2015). Nevertheless the charge modifications produced by phosphorylation of RPS6 probably have important biological roles either within the ribosome or for extra-ribosomal functions of RPS6. Indeed it was recently reported that RPS6 affects ribosomal RNA production and interacts with the HD2B histone deacetylase (Kim et al., 2014). More work is therefore needed to elucidate the role of TOR in regulating translation or development through the conserved RPS6 phosphorylation.

## Author Contributions

TD, MS, MZ, CR, LR, JH, and CM conceived the research plan and supervised the experiments, TD, EM-M, CF, MA, MD, MM, JC, and OL performed the experiments and analyzed the data, TD, CF, JH, CR, and CM wrote the article with contributions of all the authors.

## Conflict of Interest Statement

The authors declare that the research was conducted in the absence of any commercial or financial relationships that could be construed as a potential conflict of interest.

## Footnotes

1 http://pappso.inra.fr/bioinfo/xtandempipeline/

2 http://urgv.evry.inra.fr/cgi-bin/projects/CATdb/consult_expce.pl?experiment_id=302

3 http://www.arabidopsis.org/

4 http://meme-suite.org/

5 http://moulon.inra.fr/protic/tor_inactivation
