# Supplementary material for: The Arabidopsis TOR Kinase Specifically Regulates the Expression of Nuclear Genes Coding for Plastidic Ribosomal Proteins and the Phosphorylation of the Cytosolic Ribosomal Protein S6
Source: Front Plant Sci. 2016 Nov 7;7:1611. doi: 10.3389/fpls.2016.01611 (PMC5100631; doi:10.3389/fpls.2016.01611)
Supplement: Supplementary file 1 [file Presentation_1.pdf]

Supplementary Figure S1. Details of MEME analysis for Figure 4A.

****

MEME - Motif discovery tool

****

MEME version 4.11.2
For further information on how to interpret these results or to get
a copy of the MEME software please access http://meme-suite.org .
Timothy L. Bailey and Charles Elkan,
TRAINING SET
****

Sequence name	Weight	Length	Sequence name	Weight	Length
AT1G05190	1.0000	92	AT1G07320	1.0000	57
AT1G16790	1.0000	85	AT1G29070	1.0000	93
AT1G32990	1.0000	42	AT1G35680	1.0000	41
AT1G48350	1.0000	65	AT1G64510	1.0000	50
AT1G68590	1.0000	63	AT1G71720	1.0000	23
AT1G74970	1.0000	100	AT1G75350	1.0000	92
AT1G78630	1.0000	41	AT1G79850	1.0000	30
AT2G24090	1.0000	74	AT2G33450	1.0000	41
AT2G33800	1.0000	78	AT2G38140	1.0000	65
AT2G43030	1.0000	62	AT3G13120	1.0000	104
AT3G15190	1.0000	75	AT3G20230	1.0000	79
AT3G23700	1.0000	46	AT3G25920	1.0000	77
AT3G27160	1.0000	55	AT3G27830	1.0000	71
AT3G27850	1.0000	66	AT3G44890	1.0000	71
AT3G52150	1.0000	50	AT3G54210	1.0000	50
AT3G56910	1.0000	99	AT3G63190	1.0000	133
AT3G63490	1.0000	66	AT4G01310	1.0000	67
AT4G17560	1.0000	64	AT4G29060	1.0000	234
AT4G34620	1.0000	79	AT5G13510	1.0000	32
AT5G13720	1.0000	177	AT5G14320	1.0000	140
AT5G15760	1.0000	47	AT5G17870	1.0000	42
AT5G24490	1.0000	89	AT5G30510	1.0000	75
AT5G40950	1.0000	84	AT5G47190	1.0000	43
AT5G54600	1.0000	68	AT5G65220	1.0000	72

****
****

MOTIF 1 MEME width = 21 sites = 28 llr = 303 E-value = 6.5e-024

****

Multilevel consensus TCTTCTTCTTCATCCTCATTT

Motif 1 sites sorted by position p-value

```
-------------------------------------------------------------------------------
Sequence name                     Start   p-value                Site
-------------          ----- ---------         ---------------------
AT3G27160                     25  1.74e-10 CAATTTCTCT TCTTCTTCTTCTTCTTCCTCG CGACGCGTTA
AT5G65220                     43  4.28e-10 AGAACTCTGC TCTTCTTCTTCTTCCTCCGCC ATAGCTACA
AT5G14320                     66  7.14e-10 TCCGTTCAGC TCATCTTCTTCTTCTTCTTCG TCACCTCTGA
AT1G07320                     12  1.12e-08 CTTTCTCAGT CTTTCTTCTTCTTCTTCGTTG TGATAGGTCA
AT4G34620                     34  1.35e-07 AAACCTTTAC TTATCTTCTTTCTTCTCACTT CTCAGACCTC
AT3G63490                     22  1.55e-07 AGACTCGCAC TCTTCTTCTTCTTCTTTGTAC CCAGAAAGAA
AT1G75350                      3  2.95e-07         CT TCATCATCCTCATTCTCTTAT CCATCAATCG
AT1G74970                     17  4.25e-07 ACTCTGCTCG TTATCTTCTTCCTTTTTACCT GTTCAACGAA
AT1G05190                     34  5.38e-07 AACGCTCTTC TTCTTCTCTTCATCCTCCTTC TGGAGCAGAG
AT3G25920                     22  1.61e-06 CTCTCTCTAT CTATCTTCCTCAACCTTATCC TTGAAGCTTT
AT2G33800                     26  1.78e-06 GCTGGATAAG CCTTTTGCTTCATTCTCACTT CCTTCCTCAT
AT2G24090                      8  1.78e-06    ATCTTCT TCATCATCTCTTTCCTCAATC GGAGTGAGCG
```

AT3G63190	44	2.40e-06	AACAATTCGG	TCTGCTTCGTCTCCCTCCCTT	TCAATTTTCG
AT2G38140	16	2.40e-06	CACTGCCTTA	TCTTCTTCTGGATCTTCTTCT	TCGAACAAAT
AT5G40950	11	4.64e-06	AAGGAGAAGA	ACTTCTTCTTCAACCTCAGAA	AACCCTAATT
AT3G52150	14	7.85e-06	GACACTCGCA	ACTTCTTCCTTATCGACACCT	ACACTCTCCT
AT1G68590	6	8.54e-06	TGACC	ATATCTTCCTTATCCATATTC	TCACAAAATC
AT3G23700	2	1.09e-05	A	TCATTACCTTCAATCTCCGTG	GCTTTTTTGC
AT4G29060	9	1.39e-05	GAAGACTC	TTATCTCCTCTATCCACCAAG	AGAGGGAAAA
AT1G64510	15	1.75e-05	AAATTTTACT	CTCTCATCTCCTCTTTGCTTT	CACAATCTCA
AT2G43030	35	1.89e-05	CTCTCTCAGA	GTTTCTTCTCCTCTGTGAATC	TCCGACA
AT5G13720	117	2.19e-05	ACGCGCCGAT	CTTTCTTCTACTACTTGTCCT	CCGGCAATAA
AT5G47190	3	2.36e-05	AA	GGCTCTTCTTTACTCTGGTTT	CGAGTTTGGG
AT3G20230	49	4.72e-05	ACTCCGAGAG	CTTTCTTTCTCACTCTTCAAG	CTCGACGAAG
AT1G32990	17	4.72e-05	TAGCTTTCAC	TAACCTTCTTCCTTCACAGTG	AATCA
AT5G13510	5	5.37e-05	AGAA	ACAGTCTCCTCATCTTCTGAG	CAAAAAA
AT4G17560	20	8.30e-05	CTTTTCCACT	GTCTTCCCTCCATTTTCTCCG	AGTAACTGAG
AT5G24490	6	9.90e-05	TATTT	TCCACTTCCTTTCTCACTACT	TCAGTCTTCA

****

MOTIF 2 MEME width = 21 sites = 12 llr = 190 E-value = 2.1e-011

****

Motif 2 Description

Multilevel consensus GAGGGAAAAAGAAGAAGAGAG

Motif 2 sites sorted by position p-value

Sequence name	Start	p-value		Site	
AT3G27850	37	1.64e-11	CCAAAGCTTC	AGAGGAAGAAGAAGAAGAGAG	TGAGAAACA
AT5G54600	27	1.93e-11	CGGATAGGAC	GAAGGAAAAAGAAGAAGAGTG	AAAGTTGCAG
AT3G15190	31	7.67e-10	TACTAACAGA	GGGAGAAAGAGCAGAGAAGAG	AGAAGAGGAA
AT1G75350	57	8.70e-10	TCTCTATCAC	CAGGAAAAAAGAAGAAGATAG	CTCCTGAAGA
AT3G13120	57	5.05e-09	TCGAACGTTA	AGAGGAAGAAGAATAAGAGAA	ATCGATTCGT
AT1G48350	44	2.31e-08	GAGAGCGAGC	CAGAGAGAGTGAGTAAGAGAG	A
AT1G78630	11	5.37e-08	GGAAAGAGCA	GAAGAAATTTGCAGAAGAGAG	AAAAACAAAA
AT5G14320	13	2.04e-07	AAGTAGAGAC	GGAGCAAAAACAAGAGAAGCG	TTTGGATTCG
AT3G27830	43	2.34e-07	AAGAAAAACT	CAGAAGAAAAGCCAAAGAGTG	AAACAAAA
AT4G29060	57	2.85e-07	AAAATAGCAC	AGGGGAGAGACAAAATGAGAT	AAGGATTTGA
AT3G56910	28	3.24e-07	GACACAGACA	GAGAGAAGGAGAAAAAGCTTA	TCCACTATCA
AT1G71720	3	3.24e-07	CG	CGGCGGATAAGAGGAAAAAAG	

Sup Figure S2

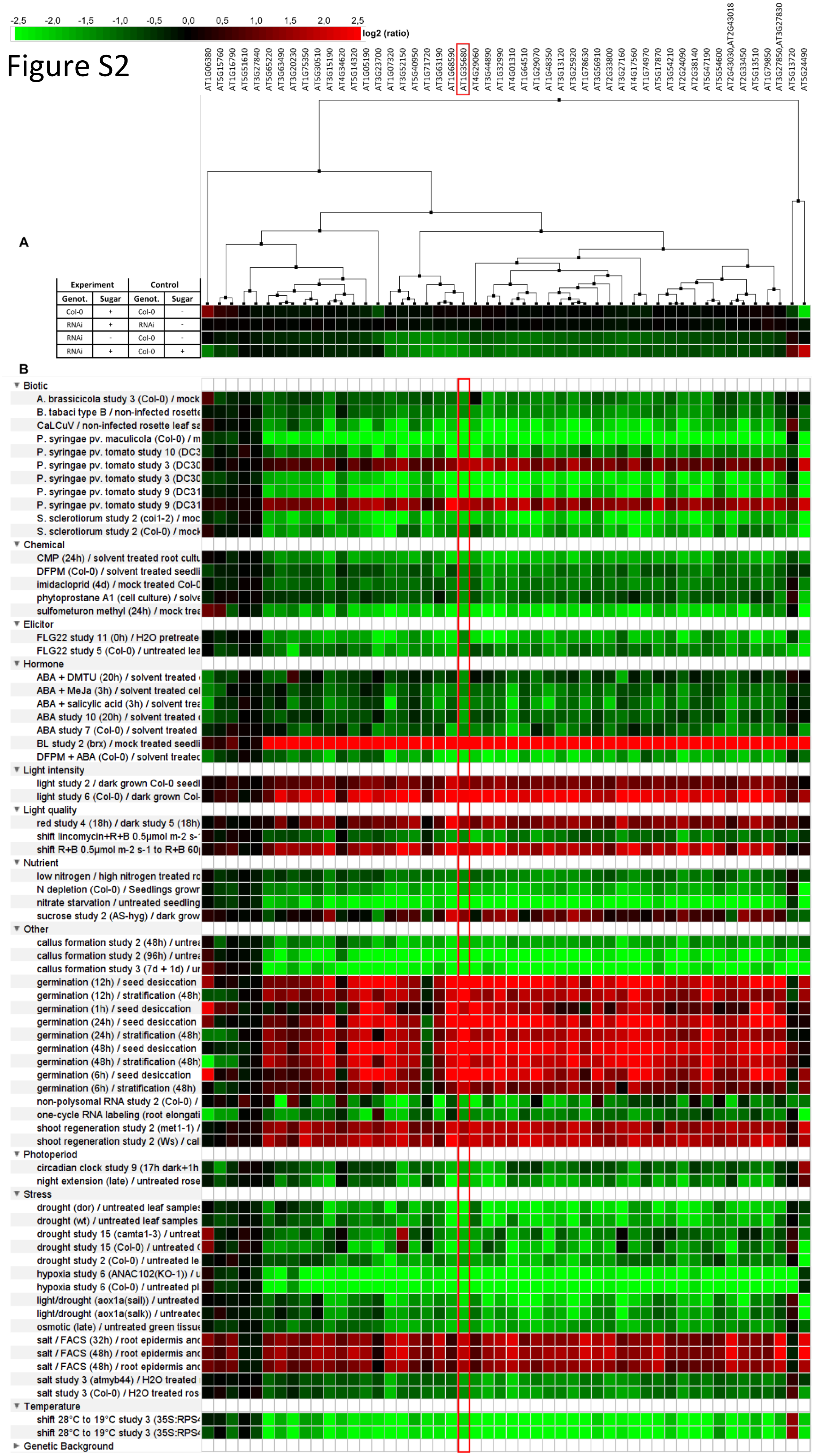

Supplementary Fig. S2: Expression profile of the chloroplastic ribosomal genes encoded by the nuclear genome. (A) Expression profile in the experiment AT-00696 (Genevestigator nomenclature, Xiong *et al*., 2013) in which seeds of an estradiol inducible TOR RNAi line or Col-0 wild-type were germinated on estradiol-containing medium (without any sugar) and, after 3 days of growth, supplemented or not with 15mM glucose, (B) Expression profile of these genes in the "perturbations" sub-group of the Genevestigator database. Experiments are grouped according to the type of treatment used. Only experiments in which the At1g35680 gene showed an absolute logarithmic fold change of 2.5 were kept.

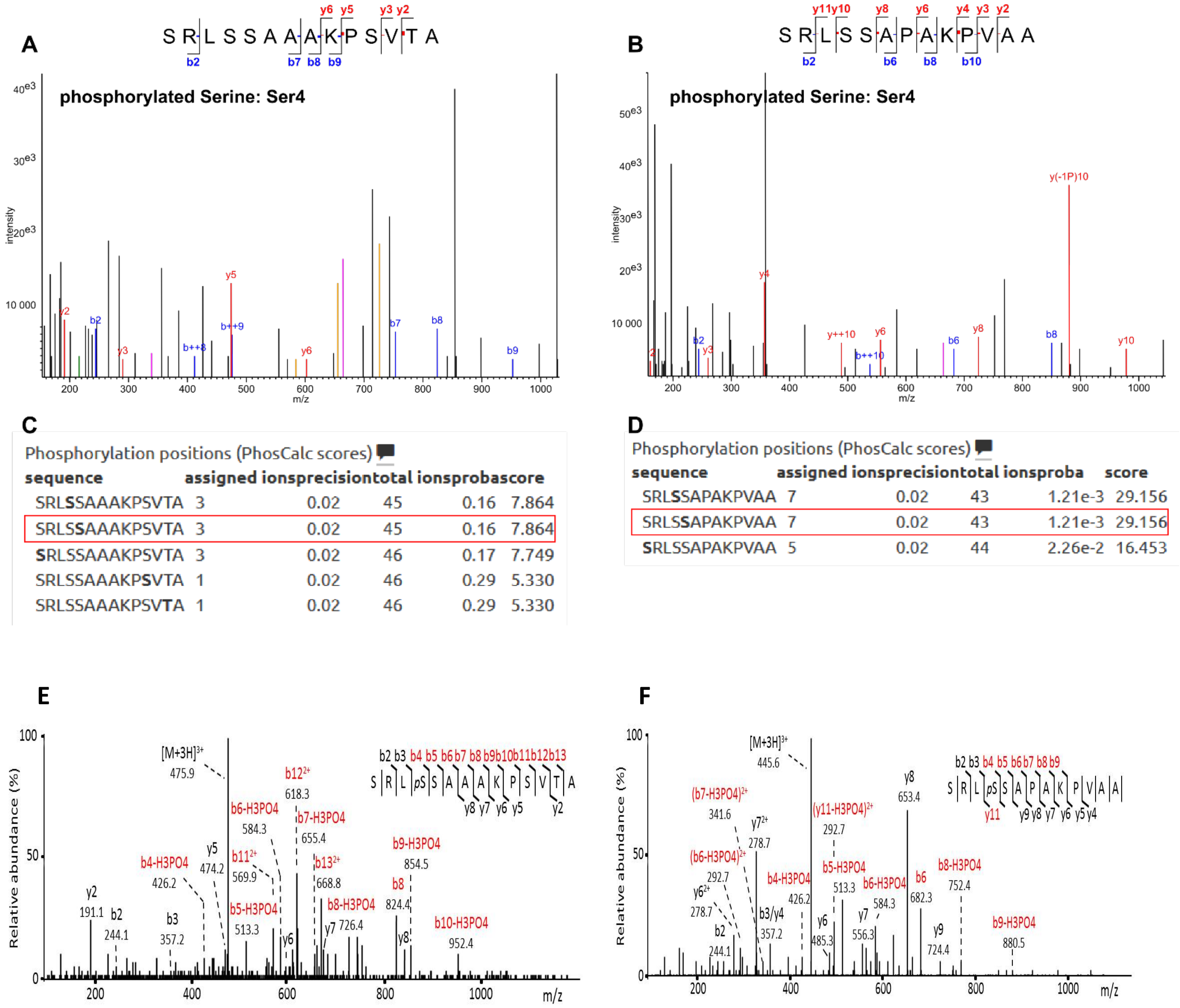

C Phosphorylation positions (PhosCalc scores)

sequence	assigned ions	precision	total ions	proba	score
SRL**S**SAAAKPSVTA	3	0.02	45	0.16	7.864
SRLS**S**AAAKPSVTA	3	0.02	45	0.16	7.864
**S**RLSSAAAKPSVTA	3	0.02	46	0.17	7.749
SRLSSAAAKP**S**VTA	1	0.02	46	0.29	5.330
SRLSSAAAKPSV**T**A	1	0.02	46	0.29	5.330

D Phosphorylation positions (PhosCalc scores)

sequence	assigned ions	precision	total ions	proba	score
SRL**S**SAPAKPVAA	7	0.02	43	1.21e-3	29.156
SRLS**S**APAKPVAA	7	0.02	43	1.21e-3	29.156
**S**RLSSAPAKPVAA	5	0.02	44	2.26e-2	16.453

Supplementary Fig. S3: Statistical analysis of the phosphorylation positions in the C-terminal peptides of the RPS6 proteins.

(A) and (B), Spectra of phosphorylated peptides of RPS6A and RPS6B (respectively) are displayed with the corresponding sequence with the b ions (from the N-terminal extremity) and the y ions (from the C-terminal extremity). The position of the phosphorylation predicted by the X!Tandem software is indicated. "-1P" represents the neutral loss of a phosphate group. "b++" and "y++" ions represent the shift due to the phosphorylation. Parental ion fragments are: SRLpSSAAAKPSVTA (m/z=713,3587, z=2) and SRLpSSAPAKPVAA (m/z=667,8453, z=2) and were identified in 2 and 5 samples respectively. (C)and (D,) Phoscalc analysis of spectra displayed in A and B, respectively, showing the statistical analysis of the phosphorylation sites predictions. (E) and (F), Spectra of phosphorylated peptides of RPS6A and RPS6B (respectively) obtained after IMAC purification of phosphorylated peptides from auxin-treated plants. Peptides are displayed with the corresponding sequence with the b ions (from the N-terminal extremity) and the y ions (from the C-terminal extremity).

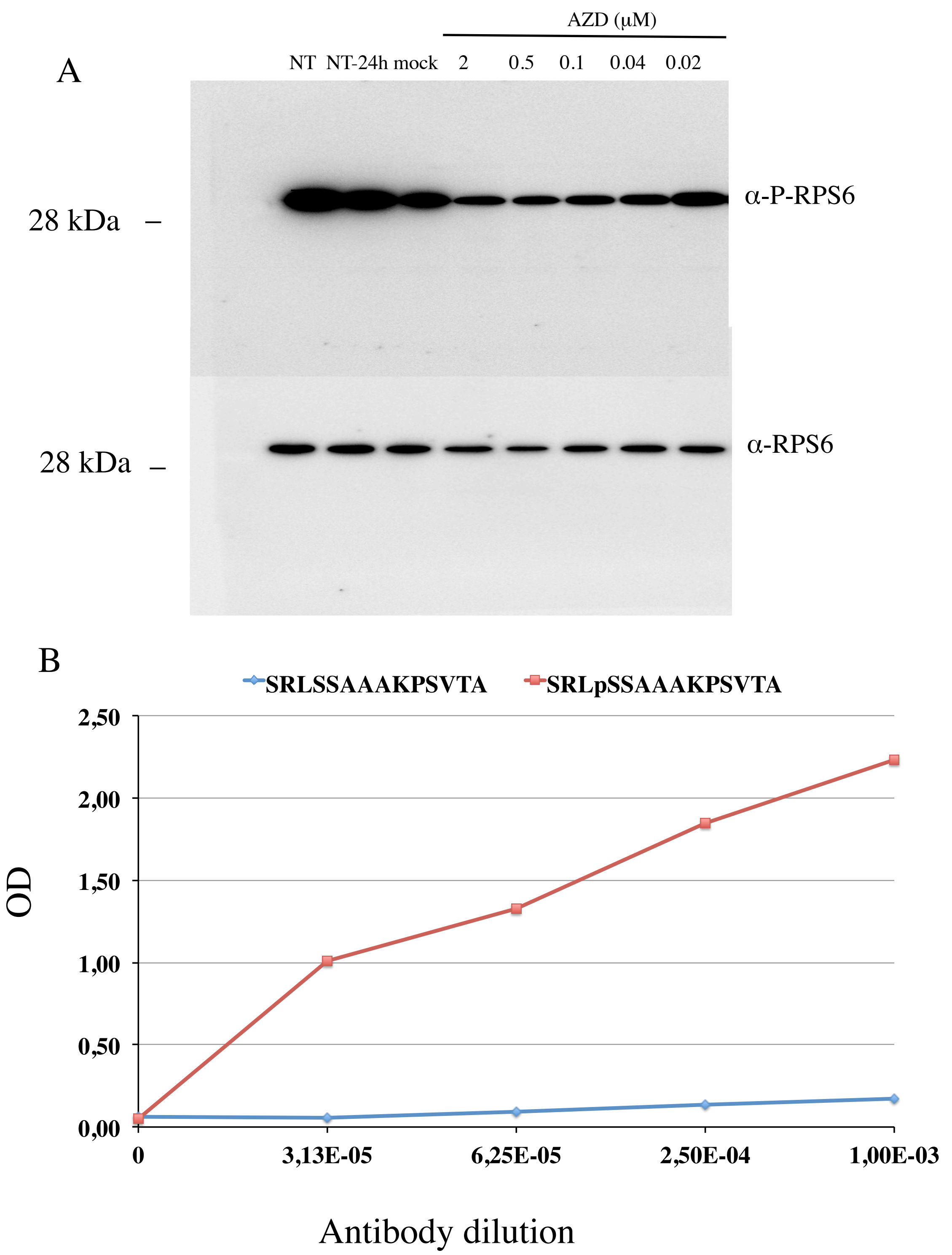

Supplementary Fig. S4: Western blot analysis of RPS6 phosphorylation.

(A), Image of the whole Western blot membrane showing the specificity of the RPS6 antibodies. Total protein extracts obtained from seedlings were separated by SDS-PAGE and blotted onto a membrane. After incubation with the phosphospecific antibody against RPS6 Ser240 (P-RPS6) or a monoclonal antibody against total animal RPS6 (RPS6), blots were revealed by a secondary antibody linked to HRP activity and imaged with a CCD camera (see Material and Methods for details).

Samples are in the same order as in Fig. 7A with a longer exposure time.

(B), ELISA test with the phosphospecific antibody against RPS6 Ser240. The phosphopeptide used for antibody production and the corresponding non-phosphorylated peptide were coated on microtitration plates. The anti-phosphopeptide crude serum was enriched on the immobilized RPS6 phosphopeptide to obtain phospho-specific antibodies which were used as primary antibodies in the ELISA test after sequential dilutions to evaluate their specificity.

OD after revelation by a secondary antibody coupled

Supplementary Table S1. Gene accessions numbers and quantitative values corresponding to Figure 3. Log_2 ratios for transcriptomic (two first columns RNAi1 and RNAi2) and translatomic (second columns RNAi1 and RNAi2) experiments and peak areas in arbitrary units for proteomic analyses (Exp1 and Exp2) are shown.

AGI	RNAi1	RNAi2	RNAi1	RNAi2	Exp1	Exp2
AT1G05190	-0,69	-0,62	-0,36	-0,45	0,21	0,50
AT1G06380	0,47		0,39	0,33		
AT1G07320					0,60	
AT1G16790	-0,38	-0,51	-0,46	-0,58		
AT1G29070	0,36	0,36	0,78	0,47		
AT1G32990	-0,58	-0,44	-0,40		0,50	0,67
AT1G35680					0,17	
AT1G35680					0,17	
AT1G48350	-0,33			-0,34	0,50	
AT1G64510						
AT1G68590	-0,45	-0,39				
AT1G71720	-0,38					
AT1G74970	-0,45	-0,31	-0,53	-0,53		0,83
AT1G75350	-0,35	-0,40	-0,33	-0,38	0,50	0,83
AT1G78630	-0,55	-0,55	-0,32		0,60	0,83
AT1G79850	-0,35	-0,30		-0,35	1,50	0,67
AT2G24090						
AT2G33450	-0,61	-0,36				
AT2G33450						
AT2G33800	-0,39	-0,44	-0,36	-0,53	0,64	
AT2G38140	-0,52	-0,35				
AT2G43030			-0,53	-0,56	0,50	1,13
AT3G13120						
AT3G15190	-0,44	-0,41		-0,30	0,17	0,50
AT3G15190				-0,30	0,17	0,50
AT3G20230						
AT3G20230						
AT3G23700	-0,67	-0,57		-0,35		
AT3G25920	-0,44	-0,38			0,54	0,56
AT3G27160	-0,44	-0,48				
AT3G27830					0,83	0,50
AT3G27840						
AT3G27850	-0,36					
AT3G44890	-0,43	-0,32			0,50	0,33
AT3G52150		-0,36				
AT3G54210						
AT3G56910						
AT3G63190	-0,47			0,35		
AT3G63490					0,38	0,58
AT3G63490					0,38	0,58
AT3G63490		-0,37			0,38	0,58
AT4G01310	-0,37				0,50	1,10
AT4G17560		-0,49		-0,54		
AT4G29060	-0,56	-0,36				
AT4G34620	-0,31	-0,32		-0,54	0,33	
AT5G13510	-0,36	-0,38			0,36	0,17
AT5G13510	-0,35	-0,34		-0,31	0,36	0,17
AT5G13720	0,56	0,49	0,91	0,84		
AT5G13720		0,35	0,55	0,55		
AT5G14320		-0,38			0,50	0,75
AT5G15760						
AT5G17870	-0,30		-0,43	-0,80		
AT5G24490	-0,38	-0,69				
AT5G24490	-0,44	-0,65	-0,39			
AT5G30510	-0,31				0,54	1,17
AT5G40950					0,50	
AT5G47190	-0,49	-0,58		-0,37	0,20	
AT5G51610						
AT5G54600					0,36	0,33
AT5G63300						
AT5G65220					0,38	0,50

Supplementary Table S2: TOR inactivation affects the ribosomal protein peptides accumulation.

Effect of TOR inactivation on the peptide quantities evaluated by the fold-change calculation (Log2(ratio)). Only the peptides found as significantly differently accumulated by Student *t*-test (*: p-value<0,05, **: p-value<0,01, ***: p-value<0,001) are shown. Negative values correspond to peptides that are down-regulated during TOR inactivation.

oxM : oxidized methionines.

Peptide sequence	Proteins	RP families	Protein paralogs				Log2(ratio)
			A	B	C	D	
TEKDVTPoxMGGFPHYGIVK	At1g43170	L3	X				-0,757*
IINSDEIQSVVNPIKK	At3g09630	L4	X				-0,419**
GVEAESIEEoxMYKK	At5g39740	L5		X			-0,599*
NYIYGGHVSNYoxMK	At3g25520, At5g39740		X	X			-0,583*
FDDKYFGK	At1g18540, At1g74050 At1g74060	L6	X	X	X		-0,320*
TEGEFFEAEKEEK	At1g18540, At1g74050 At1g74060		X	X	X		-0,497*
ANFNDKYEEYR	At2g47610, At3g62870	L7a	X	X			-0,564*
LKVPPALNQFTK	At2g47610, At3g62870		X	X			-0,512*
KoxMEVPYCIVK	At2g47610, At3g62870		X	X			-0,738*
VPPALNQFTK	At2g47610, At3g62870		X	X			-0,350*
KVEoxMLDGVTIVR	At1g33120, at1g33140	L9		X	X		-0,862*
VHPFHVLR	At1g14320, At1g26910 At1g66580	L10	X	X	X		-0,581*
ICoxMLGDAQHVEEAEK	At1g08360, At2g27530 At5g22440	L10a	X	X	X		-0,473*
KYHAFLASESVIK	At1g08360		X				-0,604*
LAPTIGISVDHR	At5g23900	L13				X	-0,403*
PTSGPLRPVVHGQTLK	At3g49010, At5g23900			X		X	-0,639**
YYEIILVDPAHNAVR	At4g16720, At4g17390	L15	X	X			-0,538*
GIVYGKPTNQGVTQLK	At4g16720, At4g17390		X	X			-0,836*
LVEFoxMTGKDDK	At5g27850	L18			X		-0,650*
YPLTTESAoxMKK	At2g39460, At3g55280	L23a	X	X			-0,638*
TPLPDVVIIHAPK	At2g31610	S3	X				-0,493*
DGYMVSSGQPTK	At3g53870, At5g35530 At2g31610		X	X	X		1,5979*
LoxMEVHGDYTAEDVGVK	At4g34670	S3a		X			-0,546*
YPDPLIKPNDTIK	At2g17360,	S4	X	X		X	-0,227*

	At5g07090<br>At5g58420						
GVSDLPGLTDTEKPR	At5g10360			X			-0,387*
KGVSDLPGLTDTEKPR	At5g10360	S6		X			-0,736*
KGENDLPGLTDTEKPR	At4g31700		X				-0,552*
HIDFALTSPFGGGRPGR	At5g15200	S9		X			-0,917*
QLVNIPSFMVR	At5g15200,<br>At5g39850		X	X			-0,257*
IQDKEGIPPDQQR	At2g47110,<br>At3g62550	S27a		X	X		-2,175***

Supplementary Table S3: Analyses of RPS6 phosphorylated peptides in extracts from *Arabidopsis* plants treated by auxin.

Accession number	RPS6 isoform	Range	Sequence	Metascore	Error, ppm	Phospho site
AT4G31700.1	S6a	237 - 250	R.SRLSSAAAKPSVTA.-	54.1	-4.80	Ser237 and Ser240
AT4G31700.1	S6a	237 - 250	R.SRLSSAAAKPSVTA.-	49.8	-0.08	Ser240
AT4G31700.1	S6a	237 - 250	R.SRLSSAAAKPSVTA.-	44.2	-1.15	Ser240
AT4G31700.1	S6a	237 - 250	R.SRLSSAAAKPSVTA.-	59.9	8.25	Ser240
AT4G31700.1	S6a	237 - 250	R.SRLSSAAAKPSVTA.-	41	0.79	Ser237, Ser240 or Ser241
AT5G10360.1	S6b	228 - 236	R.RSESLAKKR.S	47.2	-3.62	Ser231
AT5G10360.1	S6b	237 - 249	R.SRLSSAPAKPVAA.-	46.4	-2.94	Ser240
AT5G10360.1	S6b	237 - 249	R.SRLSSAPAKPVAA.-	63.8	-5.41	Ser240
AT5G10360.1	S6b	237 - 249	R.SRLSSAPAKPVAA.-	46.5	-0.83	Ser240 or Ser241
